# Supplementary material for: A long non-coding RNA targets microRNA miR-34a to regulate colon cancer stem cell asymmetric division
Source: eLife. 2016 Apr 14;5:e14620. doi: 10.7554/eLife.14620 (PMC4859802; doi:10.7554/eLife.14620)
Supplement: Figure 1—source data 1. — Tissue specimens collected from the listed CRC patients were used for analyses of Lnc34a and miR-34a expression and miR-34a promoter methylation. DOI: http://dx.doi.org/10.7554/eLife.14620.004 [file elife-14620-fig1-data1.doc]

**Figure 1-source data 1. Information of CRC patients.**

| **Patient** | **Gender** | **Age at visit** | **Stage** | **Differentiation** | **Lymph nodes** |
| --- | --- | --- | --- | --- | --- |
| P1 | M | 44**.56** | I | Moderate | N0 |
| P2 | M | 61 | I | Moderate | N0 |
| P3 | M | 85 | I | NA | N0 |
| P4 | F | 84 | I | Moderate | N0 |
| P5 | F | 65 | I | Moderate | N0 |
| P6 | M | 79 | I | NA | N0 |
| P7 | F | 88 | I | Moderate | N0 |
| P8 | F | 63 | I | Moderate | N0 |
| P9 | M | 61 | I | Moderate | N0 |
| P10 | M | 54 | I | Moderate | N0 |
| P11 | M | 85 | I | NA | N0 |
| P12 | M | 70 | I | Poor | N0 |
| P13 | M | 66 | I | NA | N0 |
| P14 | M | 81 | I | NA | N0 |
| P15 | M | 52 | I | Well | N0 |
| P16 | F | 57 | IIA | Moderate | N0 |
| P17 | F | 70 | IIA | NA | N0 |
| P18 | M | 43 | IIA | NA | N0 |
| P19 | F | 48 | IIA | Moderate | N0 |
| P20 | F | 68 | IIA | Poor | N0 |
| P21 | M | 81 | IIA | Poor | N0 |
| P22 | F | 92 | IIB | Well | N0 |
| P23 | M | 81 | IIB | Poor | N0 |
| P24 | F | 50 | IIIA | Moderate | N1 1-3 |
| P24 | M | 73 | IIIA | Well | N1 1-3 |
| P25 | F | 54 | IIIB | Moderate | N1 1-3 |
| P26 | M | 62 | IIIB | NA | N1 1-3 |
| P27 | F | 77 | IIIB | Poor | N1 1-3 |
| P28 | F | 71 | IIIC | NA | N2 >3 |
| P29 | F | 50 | IIIC | Poor | N2 >3 |
| P30 | F | 84 | IIIC | Moderate | N2 >3 |
| P31 | M | 85 | IIIC | Poor | N2 >3 |
| P32 | F | 61 | IIIC | Poor | N2 >3 |
| P33 | F | 88 | IIIC | Poor | N2 >3 |
| P34 | F | 70 | IIIC | Poor | N2 >3 |
| P35 | F | 73 | IV | Poor | N2 >3 |
| P36 | F | 73 | IV | Moderate | N1 1-3 |

| P37 | M | 66 | IV | Poor | N1 1-3 |
| --- | --- | --- | --- | --- | --- |
| P38 | M | 56 | IV | Poor | N2 >3 |
| P39 | F | 68 | IV | Poor | N2 >3 |
| P40 | F | 46 | IV | NA | N1 1-3 |
| P41 | F | 73 | IV | Poor | N1 1-3 |
| P42 | M | 37 | IV | Poor | N2 >3 |
| P43 | F | 21 | IV | Moderate | N1 1-3 |
| P45 | F | 69 | IV | Moderate | N1 1-3 |
