# Supplementary material for: A long non-coding RNA targets microRNA miR-34a to regulate colon cancer stem cell asymmetric division
Source: eLife. 2016 Apr 14;5:e14620. doi: 10.7554/eLife.14620 (PMC4859802; doi:10.7554/eLife.14620)
Supplement: Figure 5—source data 1. — DOI: http://dx.doi.org/10.7554/eLife.14620.015 [file elife-14620-fig5-data1.doc]

**Figure 5-source data 1. Potential Lnc34a-associated proteins identified by biotinylated Lnc34a pull-down and mass spectrometry.**

| **Protein accession #** | **Protein description** | **Mass of protein** | **# of peptide matching** | **Protein coverage (%)** |
| --- | --- | --- | --- | --- |
| gi5031857 | lactate dehydrogenase A | 43596 | 74 | 19 |
| gi332864 | pyruvate kinase 3 isoform 1 | 67178 | 61 | 18.4 |
| gi315429 | chaperonin | 73562 | 35 | 11.2 |
| gi545355 | ras-related nuclear protein | 28933 | 14 | 9.7 |
| gi13128860 | Histone Deacetylase 1 (HDAC1) | 55130 | 9 | 9.7 |
| gi488543 | heat shock 70 protein 1B | 81954 | 34 | 8.6 |
| gi600585 | prohibitin 2 (PHB2) | 37859 | 4 | 8.51 |
| gi59270 | splicing factor, arginine/serine-rich 1 isoform 1 | 29904 | 2 | 8.5 |
| gi892741 | cytokine induced apoptosis inhibitor 1 | 40777 | 4 | 8.3 |
| gi45505 | tumor-associated calcium signal transducer 1 precursor | 41981 | 4 | 7.42 |
| gi12751473 | DNA (cytosine-5)-methyltransferase 3A isoform a (Dnmt3a) | 101585 | 11 | 6.1 |
| gi517461 | nucleosome assembly protein 1-like 4 | 50530 | 12 | 5.6 |
| gi482558 | protein kinase C substrate 80K-H isoform 1 | 68149 | 6 | 4.33 |
| gi213611 | Na+/K+ -ATPase alpha 1 subunit isoform a proprotein | 127198 | 14 | 3.9 |
| gi45505 | tumor-associated calcium signal transducer 1 precursor | 41981 | 4 | 7.42 |
